# A Self‐Supervised Approach for Hainan Gibbon Call Separation in Passive Acoustic Monitoring

**DOI:** 10.1002/ece3.74542

**Published:** 2026-10-22

**Authors:** Jiali Zi, Nuo Xu, Yanqi Dong, Yi Xia, Weilin Wang, Feixiang Chen

**Affiliations:** ^1^ School of Information Science and Technology Beijing Forestry University Beijing China; ^2^ Hebei Key Laboratory of Smart National Park Beijing China

**Keywords:** bioacoustics, call separation, deep learning, passive acoustic monitoring, self‐supervised learning

## Abstract

Passive acoustic monitoring (PAM) provides an effective approach for ecological research and endangered species conservation. However, automated analysis of PAM recordings from the critically endangered Hainan gibbon ( *Nomascus hainanus* ) remains challenging due to environmental noise and the lack of paired clean reference recordings. Supervised source separation methods often require extensive annotated datasets, limiting their applicability to real‐world ecological recordings. To address these challenges, this study proposes MC‐RemixIT, a self‐supervised source separation approach designed for Hainan gibbon calls under complex acoustic conditions. Building on RemixIT, MC‐RemixIT introduces a lightweight call enhancement module with attention mechanisms, signal cross‐correlation constraints to improve target–noise separation, and multiple training constraints to enhance separation robustness. The proposed approach operates directly on raw waveforms and does not require fully clean reference signals during training. Experimental results demonstrate that MC‐RemixIT consistently improves separation performance compared with RemixIT and bioacoustic denoising frameworks. Moreover, downstream call presence detection experiments show that target‐call estimates generated by MC‐RemixIT improve detection performance across multiple deep learning architectures, indicating enhanced usability of the separated signals for automated acoustic analysis. MC‐RemixIT provides a practical self‐supervised solution for improving wildlife acoustic recordings in noisy environments and supports large‐scale bioacoustic monitoring of endangered species.

## Graphical abstract

The automated analysis of PAM recordings for the critically endangered Hainan gibbon ( *Nomascus hainanus* ) remains significantly challenging due to complex ambient noise and the scarcity of clean annotated datasets. We proposed MC‐RemixIT, a self‐supervised approach tailored for complex environments, designed to effectively separate Hainan gibbon calls without ground‐truth clean data.

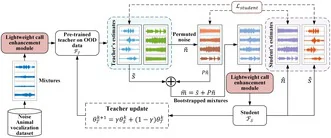

## 1 Introduction

Scientific monitoring and effective conservation of endangered species are central themes in ecology and conservation biology (Wang et al. 2025). Primates have long been a focus of research due to their complex social structures and behavioral diversity (Clink et al. 2025). The Hainan gibbon ( *Nomascus hainanus* ) is a critically endangered primate species endemic to China and currently survives only in Bawangling in Hainan Tropical Rainforest National Park (Fan 2017; Ren et al. 2022). Given its restricted habitat and small population size, continuous monitoring of their survival and population dynamics is essential for maintaining regional ecological balance and biodiversity (Deng et al. 2017). However, traditional visual surveys are often constrained by complex terrain, dense vegetation, adverse weather, and human interference, particularly in highly structured ecosystems such as tropical rainforests (Gibb et al. 2019). In contrast, acoustic monitoring is non‐invasive and enables large‐scale, long‐term remote monitoring of species even in visually challenging environments (Hao et al. 2025; Symes et al. 2022). With advances in recording devices and automated acoustic analysis techniques, acoustic monitoring has become an increasingly important tool for studying and protecting vocally active animals (Cominelli et al. 2024; Lahoz‐Monfort and Magrath 2021). Specifically, passive acoustic monitoring (PAM), which leverages autonomous recording units to continuously collect animal sounds, provides valuable data for monitoring population dynamics, investigating behavioral patterns, optimizing management, and assessing individual welfare (Kershenbaum et al. 2025; Lauha et al. 2022).

Although PAM enables continuous, long‐term data collection for biodiversity assessment, the efficient extraction, annotation, and analysis of target calls from massive datasets remain major challenges in ecoacoustics (Jeantet and Dufourq 2023). Long‐term continuous monitoring generates large amounts of redundant recordings unrelated to research objectives, from which extracting target signals requires substantial time and expert knowledge (Rugg et al. 2025). During peak call periods, such as the dawn chorus, multiple species often call simultaneously, leading to high spectro‐temporal overlap among sound sources (Denton et al. 2021). This phenomenon substantially increases the complexity of source separation and single‐species identification. Furthermore, field recordings are highly susceptible to interference from persistent biotic noise (e.g., insect stridulation), abiotic noise (e.g., wind and rain), and anthropogenic disturbances. Such background noise can be closely coupled with the target call, often masking critical acoustic features and rendering traditional analysis methods unreliable under low signal‐to‐noise ratio (SNR) conditions (Nanni et al. 2020).

Rapid advances in deep learning have enabled powerful tools for automated processing and analysis of ecoacoustic data. Deep learning models, including convolutional neural networks (CNNs) and end‐to‐end architectures, have demonstrated strong performance in complex acoustic scenes. For instance, BioCPPNet is a lightweight U‐Net‐based architecture for bioacoustic source separation, which has demonstrated effective separation of calls from macaques, bottlenose dolphins, and Egyptian fruit bats (Bermant 2021). Similarly, Xie et al. proposed BACPPNet, an end‐to‐end model based on Deeplabv3plus for bird song separation (Xie et al. 2024), while Wang et al. introduced a pix2pix GAN‐based spectrogram transformation method validated on eight bird species (Wang et al. 2025). Despite these advancements, most of these approaches rely on supervised learning and require clean target signals as ground‐truth references. Furthermore, their performance may depend on the similarity between the acoustic characteristics of the training and deployment environments. However, obtaining a fully isolated target call is often impractical in real‐world ecological monitoring. Some architectures assume a fixed number of sound sources, which may not adequately address the dynamic nature of field soundscapes. While synthetic data simulation is a common strategy (Scheibler et al. 2018), synthetic samples often struggle to replicate the complex, non‐stationary noise structures of natural environments. This discrepancy frequently results in domain mismatch, leading to a significant decline in separation performance during field application (Farahani et al. 2021).

Therefore, reducing the reliance on ground‐truth clean labels by exploring unsupervised or weakly supervised separation methods tailored for complex ecological soundscapes has become a pivotal research direction in bioacoustics. Recent studies have attempted to address this challenge without relying on clean reference signals. For instance, ORCA‐PARTY achieved automated separation of overlapping orca vocalizations (Bergler et al. 2022), its over‐reliance on synthetic training data may limit generalization to real‐world field recordings. Denton et al. applied the MixIT algorithm for unsupervised birdsong separation (Denton et al. 2022), but the approach can be limited by a fixed number of output sources and the risk of over‐separation. PriorDeNet has been applied to marine mammal denoising (Zhu et al. 2023), its performance depends heavily on the quality of the intermediate datasets. Nadiminti et al. integrated MixIT with graph attention for species classification in marine soundscapes (Nadiminti et al. 2025), but this approach struggles to achieve effective separation when background noise dominates the target signals. The Biodenoising framework uses speech enhancement models to generate pseudo‐clean labels to mitigate data scarcity (Miron et al. 2025), but its architectural inductive bias toward human speech can attenuate or even eliminate the critical high‐frequency features inherent to animal vocalizations.

Moreover, existing studies have primarily focused on birds and marine mammals, whereas source separation studies for primates remain limited. Although these studies have reported encouraging results, their methods were developed for specific species and monitoring tasks. Compared with those of birds and marine mammals, Hainan gibbon calls are generally characterized by longer durations and prominent harmonic structures (Deng et al. 2014; Dufourq et al. 2021). In addition, Hainan gibbon calls are often recorded in acoustically complex tropical rainforest soundscapes, increasing the difficulty of extracting target calls from field recordings. These challenges highlight the need to develop methods for separating Hainan gibbon calls to better support PAM.

Recent advances in self‐supervised learning have provided an effective alternative to supervised source separation (Zhu et al. 2023). RemixIT is a teacher‐student self‐supervised framework for source separation (Tzinis, Adi, et al. 2022). It iteratively optimizes the student model using pseudo‐targets generated by the teacher model, allowing the model to learn directly from mixed recordings without clean reference signals. This enables the framework to be trained directly on real‐world mixtures, making it particularly suitable for ecological monitoring, where fully clean target calls and isolated environmental noise recordings are rarely available. Building upon this framework, this study proposes MC‐RemixIT, a self‐supervised source separation approach designed for complex acoustic scenarios involving Hainan gibbon calls. Operating directly on raw time‐domain waveforms, this approach enables the effective separation of target calls from complex background noise without requiring ground‐truth pure signals. In this study, source separation experiments were conducted on a real‐world Hainan gibbon call dataset, and performance was evaluated using visual results and the Deep Noise Suppression Mean Opinion Score (DNSMOS) metric (Reddy et al. 2022). To further assess its practical utility, a downstream call presence detection task was constructed to quantify the improvement in detection performance achieved through separation‐based preprocessing.

## 2 Methods

### 2.1 Data Sources

The Hainan gibbon recordings used in this study were primarily sourced from two previously published studies. One subset of the data was obtained from the Hainan gibbon individual call dataset (Wang Dataset), which was collected in Bawangling in Hainan Tropical Rainforest National Park, Hainan, China (Wang 2024). The dataset was mainly collected from September 2021 to December 2022, with continuous recordings conducted daily from 06:00 to 18:00. The audio sampling rate was 48 kHz. Another portion of the data was obtained from the Hainan gibbon call detection dataset collected by Dufourq et al. (Dufourq Dataset), which was collected in Bawangling National Nature Reserve, Hainan, China (Dufourq et al. 2021). This dataset was collected between March and August 2016. The recording devices were configured to start recording at sunrise each day and to operate continuously for eight hours. Most recordings had a sampling rate of 9600 Hz. All audio files were stored in WAV format. Both datasets were derived from long‐term PAM recordings and capture the complex soundscape of tropical rainforests, including wind, rain, and other animals' sounds. These characteristics provide representative data for evaluating the applicability and robustness of the proposed method in real‐world ecological monitoring scenarios.

### 2.2 Separation Data Processing

To construct a standardized data format compatible with the model input, the raw audio data were uniformly preprocessed. All recordings were first segmented using a sliding window with a 10 s window length and a 1 s hop size. Following the methodology of Dufourq et al. (2021), these parameters were chosen to balance temporal contextual information with increased sample size, thereby enhancing the model's capacity to characterize non‐stationary acoustic events. To reduce the potential influence of multi‐channel recordings on feature distribution, all audio files were converted to mono format (Bergler et al. 2019; Stowell et al. 2019).

Since the audio recordings used in this study were collected from multiple sources, the original sampling rates varied among datasets. Therefore, all audio signals were resampled to a common sampling rate. The calls of Hainan gibbons exhibit acoustic energy predominantly concentrated in the low‐frequency range, typically below 2 kHz (Deng et al. 2014; Dufourq et al. 2021). A sampling rate of 16 kHz was therefore considered sufficient to preserve the essential acoustic characteristics of Hainan gibbon calls. In addition, the RemixIT framework used in this study was originally developed for speech enhancement tasks, for which 16 kHz is a commonly adopted sampling rate in speech processing applications (Panayotov et al. 2015; Tzinis, Adi, et al. 2022). This choice ensures consistency in model input representation while reducing computational complexity. Therefore, considering the acoustic characteristics of the target species, dataset consistency, and computational efficiency, all audio signals were uniformly resampled to 16 kHz.

Following preprocessing, separate datasets were constructed for teacher and student model training. Five hundred Hainan gibbon call segments with relatively low background noise were selected from the preprocessed Wang dataset to construct the teacher training dataset. These call segments were mixed with randomly selected human activity noise at different signal‐to‐noise ratios (SNR) to construct the teacher model training set, generating 4000 mixed samples. The human activity noise was sourced from the WHAM! Noise dataset (Wichern et al. 2019), from which 4000 audio samples were randomly selected to construct the noise library used for teacher model training.

The student model training data were obtained from the preprocessed Dufourq dataset, which contains field recordings with substantially more background interference than the teacher dataset. To simulate the complex situation in which individuals of the same species produce overlapping calls in natural environments, some samples were randomly paired and mixed to generate additional composite source samples, improving the model's robustness under multi‐source scenarios. Dataset partitioning was performed at the recording level rather than the clip level. All 10‐s segments derived from the same original recording were kept within the same subset to avoid potential information leakage among the training, validation, and testing sets. The final teacher dataset contains 4000 training samples, 500 validation samples, and 500 test samples. The student dataset contains 10,000 training samples, 1000 validation samples, and 1000 test samples.

### 2.3 Detection Data Processing

Based on data resources from two previously published studies (Dufourq et al. 2021; Wang 2024), a Hainan gibbon call presence detection dataset was constructed in this study. The audio segments used for training the source separation models were excluded from the validation and testing subsets of the downstream detection dataset. All raw recordings were converted to mono, uniformly resampled to 16 kHz, and segmented into 10‐s audio segments. Each segment was labeled based on the following criteria. (a) “presence”, the segment contained at least one target phrase; (b) “absence”, no target phrase was present; (c) “partial presence”, only part of a target phrase was included. To accommodate the input requirements of deep learning models, log‐Mel spectrograms were extracted from the segments as two‐dimensional acoustic representations.

The overall composition of the downstream detection dataset is illustrated in Figure 1. Figure 1a provides an overview of the dataset partition by showing the number of presence, partial presence, and absence samples in the training, validation, and testing sets. To further characterize the contribution of different recording sources, Figure 1b presents the number of samples derived from the Dufourq and Wang datasets in each partition. In addition, Figure 1c provides a more detailed breakdown of the dataset by showing the source distribution within each detection category, including presence, partial presence, and absence samples. For completeness, the detailed composition of the downstream detection dataset by recording source, subset, and detection category is provided in Table S1. The two recording sources contribute unequally across subsets, reflecting the recording‐level partitioning adopted to prevent information leakage between subsets, as well as differences in the composition of the two recording campaigns. As detailed in Table S1, the Wang test subset is comparatively small and contains no absence samples.

**FIGURE 1:**
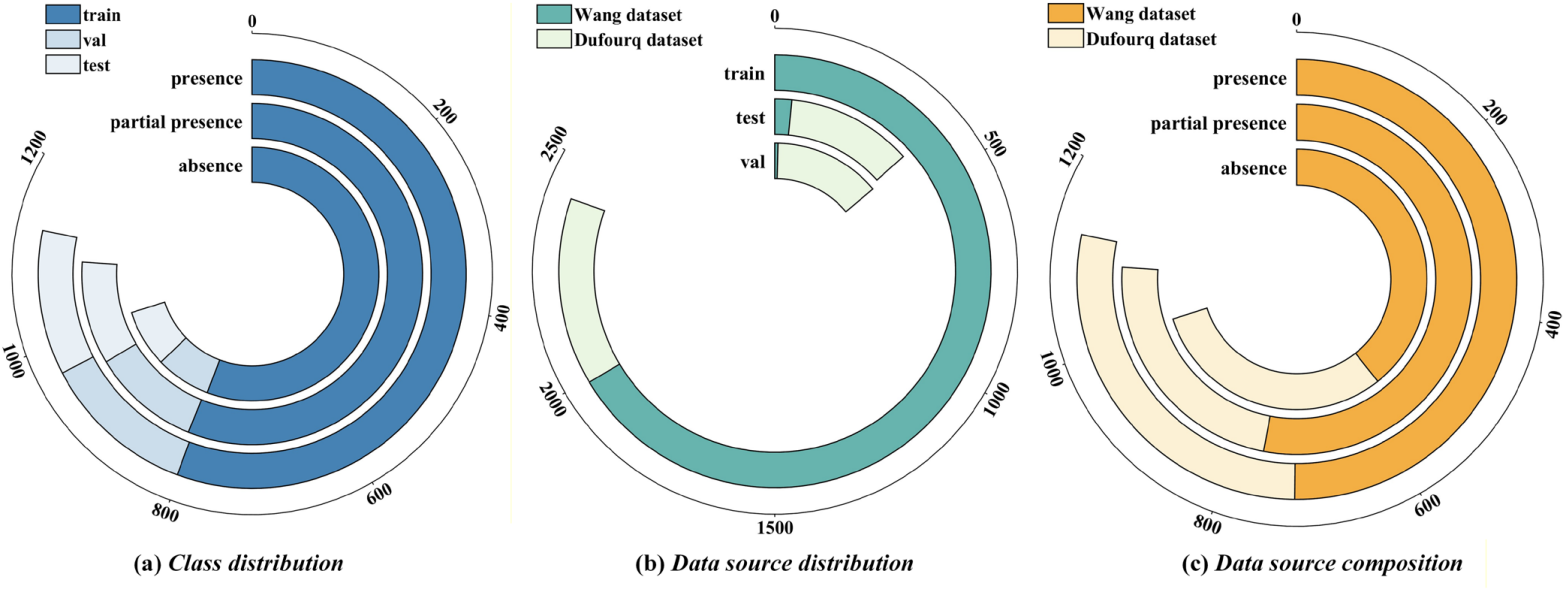
Composition of the downstream detection dataset. (a) Number of samples in the Presence, Partial Presence, and Absence categories across the training, validation, and testing sets. (b) Proportion of samples originating from the Wang and Dufourq datasets within the training, validation, and testing partitions. (c) Distribution of Presence, Partial Presence, and Absence samples across different data sources.

### 2.4 The Proposed Method

To address the challenges posed by severe overlap between Hainan gibbon calls and background noise in complex natural soundscapes, together with the lack of fully clean reference recordings, this study proposes an improved multi‐constraint cross‐correlation separation approach based on the self‐supervised learning framework RemixIT. The approach operates directly on raw time‐domain waveforms and employs a teacher‐student framework to effectively separate target species calls from background noise without fully clean reference signals. First, a lightweight call enhancement module incorporating an attention mechanism is constructed to enhance the perception of target species' call characteristics without significantly increasing computational load. Second, a signal cross‐correlation constraint is introduced during training to encourage greater acoustic separation between target calls and background noise. Finally, a multi‐constraint loss integrating spectral flatness and energy consistency is introduced to provide additional optimization constraints during student training. The overall architecture of the proposed framework is shown in Figure 2.

**FIGURE 2:**
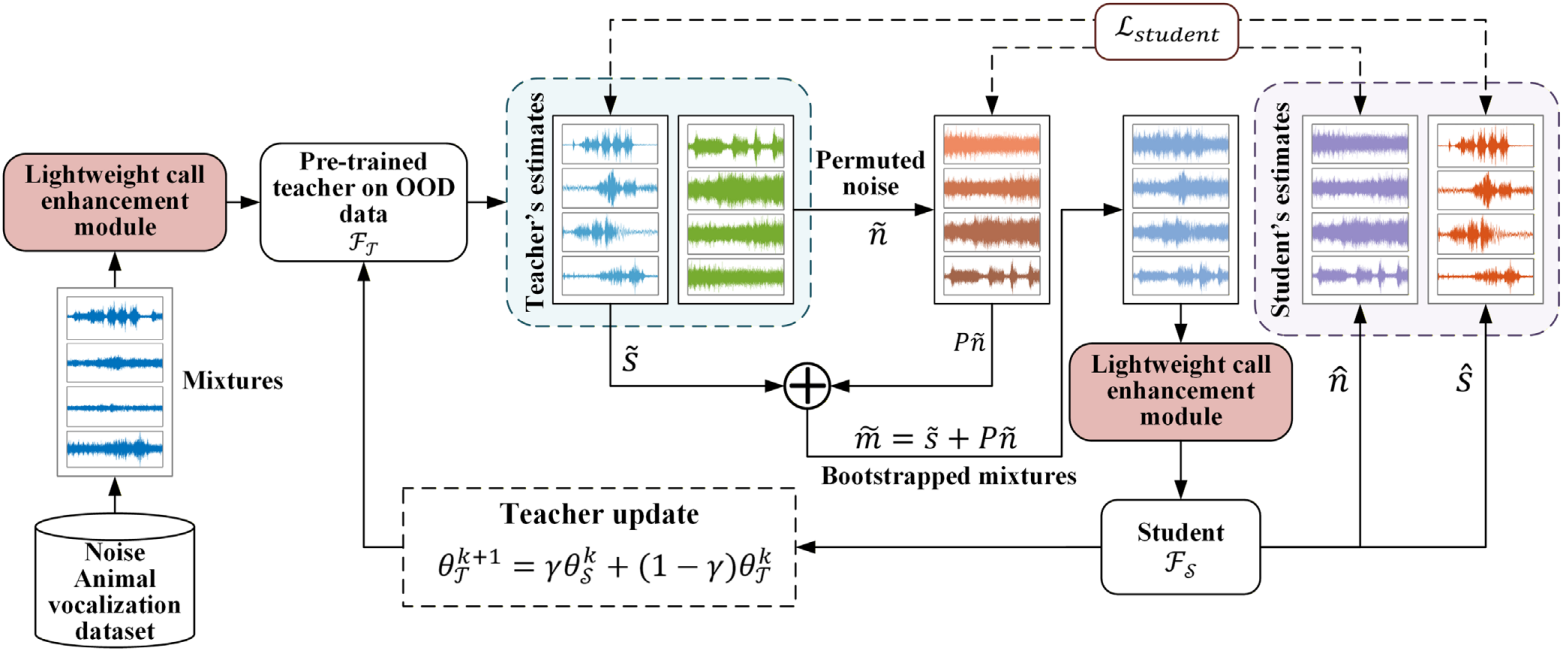
Architecture of MC‐RemixIT. OOD denotes out‐of‐domain.

#### 2.4.1 Backbone Network

In bioacoustics research, traditional deep learning models typically rely on paired clean signals and noise samples for supervised training. However, in real‐world field monitoring, obtaining clean call recordings that are representative of natural acoustic conditions is often impractical. Although unsupervised methods such as MixIT have been proposed to reduce labeling requirements via the mixture of mixtures strategy, they still assume the availability of in‐domain independent noise samples. This assumption is often difficult to satisfy in dynamic and complex ecological acoustic environments. To overcome this limitation, Tzinis et al. proposed RemixIT (Tzinis, Adi, et al. 2022). RemixIT adopts a teacher‐student self‐training mechanism that does not rely on fully clean samples or independent noise data. Instead, it leverages real mixture recordings for effective training and continuous performance improvement. This approach is particularly suitable for ecological acoustic scenarios where noise distributions are unknown and labeled data are scarce.

The core of RemixIT lies in bootstrapped remixing and continuous self‐training. It consists of a teacher model $F_{T}$ and a student model $F_{S}$. The teacher model $F_{T}$ is trained on out‐of‐domain (OOD) data. During each training epoch, the teacher model separates the input mixture signal m into an estimated target call $\tilde{s}$ and an estimated background noise $\tilde{n}$,

(1) $$\left(\tilde{s},\tilde{n}\right)=F_{T}\left(m;\theta_{T}^{\left(k\right)}\right)$$

$\theta_{T}^{\left(k\right)}$denotes the parameters of the teacher model $F_{T}$ at the *k*th training epoch. The estimated $\tilde{s}$ and $\tilde{n}$ are used as pseudo‐targets for training the student model. To construct bootstrapped training samples, the estimated noise $\tilde{n}$ is randomly permuted within each batch to obtain a permuted noise $P\tilde{n}$. The estimated call $\tilde{s}$ is then mixed with the permuted noise $P\tilde{n}$ to form a new bootstrapped mixture signal $\tilde{m}$,

(2) $$\begin{array}{c} \tilde{m}=\tilde{s}+P\tilde{n}\; \end{array}$$

The student model $F_{S}$ takes the bootstrapped mixture signal $\tilde{m}$ as input and predicts the pseudo targets $\tilde{s}$ and $P\tilde{n}$ generated by the teacher model. Through the remixing process, the student model learns to consistently extract the target call under novel interference conditions.

(3) $$\begin{array}{c} \left(\hat{s},\hat{n}\right)=F_{S}\left(\tilde{m};\theta_{S}^{\left(k\right)}\right)\; \end{array}$$

Training is performed using a signal‐level loss function L that minimizes the discrepancy between the student's estimates and the teacher's pseudo‐targets.

(4) $$\begin{array}{c} L_{\text{RemixIT}}=\sum_{b=1}^{B}\left[L\left({\hat{s}}_{b},{\tilde{s}}_{b}\right)+L\left({\hat{n}}_{b},{\left[P\tilde{n}\right]}_{b}\right)\right]\; \end{array}$$

where *B* denotes the batch size. An exponential moving average (EMA) teacher is used to synchronize the teacher and student models, where the EMA momentum coefficient is set to γ=0.01, following the default configuration in the original RemixIT framework. This setting is adopted throughout all experiments without further tuning.

(5) $$\begin{array}{c} \theta_{T}^{k+1}=\gamma \theta_{S}^{k}+\left(1-\gamma \right)\theta_{T}^{k}\; \end{array}$$

In this study, the separation model adopts the SUccessive DOwnsampling and Resampling of Multi‐Resolution Features (SuDoRM‐RF) architecture (Tzinis, Wang, et al. 2022).

#### 2.4.2 Lightweight Call Enhancement (LCE) Module

In a self‐supervised learning approach, the teacher model's initial performance directly determines the quality of the pseudo‐labels. Since field‐collected Hainan gibbon call data typically lack fully clean reference recordings of the target species, the teacher model cannot be trained under fully supervised conditions. Although existing RemixIT methods typically rely on speech datasets for out‐of‐domain teacher training, speech signals differ significantly from animal calls in their time–frequency characteristics and harmonic structure. Directly using speech datasets to train the teacher model may introduce feature mismatches, thereby affecting separation performance. Based on these considerations, this study constructs out‐of‐domain teacher training data by mixing target calls with human activity noise.

To enhance the representation of target species' call structures in both teacher and student models and reduce noise interference during training, this study incorporates a lightweight call enhancement module at the front end of both separation models. The module integrates an attention mechanism to adaptively enhance discriminative features of target calls without significantly increasing computational complexity, thereby providing more stable inputs for subsequent pseudo‐label generation and self‐supervised separation learning. The overall architecture of the module is shown in Figure 3.

**FIGURE 3:**
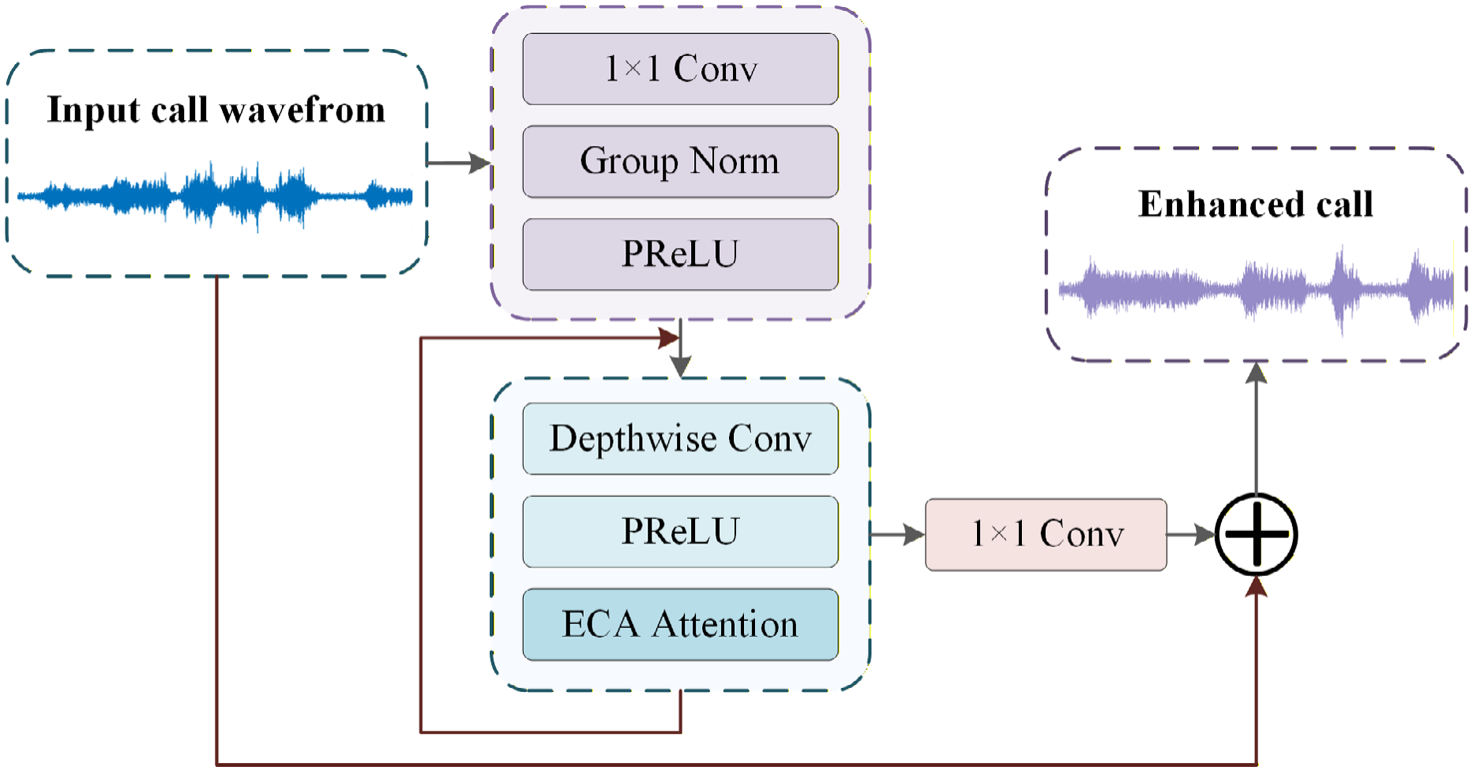
Architecture of the LCE module.

The module includes wide receptive field feature extraction, attention‐based depthwise separable convolution blocks, and global residual processing. First, the input time‐domain signal is mapped from a single‐channel waveform to a higher‐dimensional feature space via a 1D convolution layer. The convolution layer uses a large kernel to capture local acoustic features of the target calls. Normalization and PReLU activation are then applied to enhance representation for signals with varying amplitudes, producing stable and discriminative low‐level features. The module then stacks several depthwise separable convolution blocks for feature refinement. Each block consists of a depthwise separable convolution layer, PReLU activation, and a lightweight Efficient Channel Attention (ECA) (Q. Wang et al. 2020). Finally, the processed features are mapped back to the single‐channel waveform via a 1 × 1 1‐D convolution layer and combined with the original signal through a residual connection. This residual strategy enables explicit learning of noise suppression while retaining informative characteristics of the original waveform.

#### 2.4.3 Signal Cross‐Correlation Constraint

To further decouple the Hainan gibbon calls from the noise components, a signal cross‐correlation $L_{\text{corr}}$ was introduced in this study. Its objective is to minimize the statistical correlation between the estimated call and background noise (Yoo and Han 2009). Accounting for potential temporal alignment offsets, this study computes the complete cross‐correlation sequence in the frequency domain and penalizes its normalized peaks. This enhances the model's ability to separate the target calls from background noise during feature extraction, thereby generating higher‐quality pseudo‐targets in the RemixIT bootstrapping process.

To improve the separation performance of the model under self‐supervised learning, a teacher model training loss $L_{\text{teacher}}$ was defined in this study. This loss function is formulated as a weighted combination of $L_{\text{RemixIT}}$ and $L_{\text{corr}}$ to integrate the primary separation objective with the cross‐correlation constraint.

(6) $$\begin{array}{c} L_{\text{teacher}}=L_{\text{RemixIT}}+\alpha L_{\text{corr}}\; \end{array}$$

where α controls the strength of the decoupling constraint and is set to 0.2.

(7) $$\begin{array}{c} L_{\text{corr}}=\frac{1}{B}\sum_{b=1}^{B}\frac{\max_{a}\left|R_{s,n}^{\left(b\right)}\left[a\right]\right|}{\sqrt{{\left\|s^{\left(b\right)}\right\|}_{2}^{2}\cdot {\left\|n^{\left(b\right)}\right\|}_{2}^{2}}+\epsilon }\; \end{array}$$

And, $R_{s,n}^{\left(b\right)}\left[a\right]$ denotes the cross‐correlation function computed in the frequency domain.

(8) $$\begin{array}{c} \bar{s}\left(t\right)=s\left(t\right)-\frac{1}{T}\sum_{t=1}^{T}s\left(t\right),\bar{n}\left(t\right)=n\left(t\right)-\frac{1}{T}\sum_{t=1}^{T}n\left(t\right)\; \end{array}$$

(9) $$\begin{array}{c} R_{s,n}=F^{-1}\left\{F\left(\bar{s}\right)\cdot F{\left(\bar{n}\right)}^{*}\right\}\; \end{array}$$

where $F\left(\cdot \right)$ denotes the Fourier transform, ${\left(\cdot \right)}^{*}$ represents the complex conjugate, and $F^{-1}\left(\cdot \right)$denotes the inverse Fourier transform. The $\bar{s}$ and $\bar{n}$ denote the mean‐centered estimated call and noise, respectively. A larger cross‐correlation value indicates higher similarity and lower independence, whereas a smaller value implies lower similarity and greater independence.

#### 2.4.4 Multi‐Constrained Training Strategy

Because Hainan gibbon calls exhibit complex time–frequency structures, diverse calling patterns, and frequent overlap with natural background sounds, optimizing the model using a single loss function is insufficient to effectively constrain the source separation process. Therefore, a multi‐constraint joint training strategy is proposed by combining spectral flatness and energy consistency during student model training. These complementary constraints provide additional supervision for model optimization and improve the stability and reliability of source separation in complex acoustic environments. The overall objective function of the student model is defined as follows:

(10) $$\begin{array}{c} L_{\text{student}}=L_{\text{teacher}}+\omega L_{\mathrm{En}}+\upsilon L_{\mathrm{Sp}}\; \end{array}$$

where ω=0.1, υ=0.05. The energy consistency loss $L_{\mathrm{En}}$ constraints the total energy of the separated signals to remain consistent with that of the input mixture, thereby reducing large deviation in reconstructed signal energy during optimization. This constraint helps maintain a stable training process and prevents abnormal energy redistribution during self‐supervised source separation (Tan and Jiang 2018; Wisdom et al. 2019). It is formulated as

(11) $$\begin{array}{c} L_{\mathrm{En}}=\left|\frac{1}{T}\sum_{t=1}^{T}x{\left(t\right)}^{2}-\frac{1}{T}\sum_{t=1}^{T}{\left(\sum_{k=1}^{K}{\hat{s}}_{k}\left(t\right)\right)}^{2}\right|\; \end{array}$$

Spectral flatness measures the uniformity of a signal's spectral distribution and is calculated as the ratio of the geometric mean to the arithmetic mean of the magnitude spectrum (Dubnov 2004; Zamanian and Pourghassem 2017). During student model training, the spectral flatness constraint $L_{\mathrm{Sp}}$ encourages the preservation of meaningful spectral characteristics while reducing unnecessary spectral fluctuations in the separated signals. It is defined as

(12) $$\begin{array}{c} L_{\mathrm{Sp}}=\text{mean}\left(\text{Flatness}\left(t\right)\right)\; \end{array}$$

(13) $$\begin{array}{c} \text{Flatness}\left(t\right)=\frac{\exp\left(\frac{1}{F}\sum_{f=1}^{F}\log\left(\left|X\left(f,t\right)\right|+\epsilon \right)\right)}{\frac{1}{F}\sum_{f=1}^{F}\left(\left|X\left(f,t\right)\right|+\epsilon \right)}\; \end{array}$$

### 2.5 Evaluation

Since field recordings of animal calls typically lack clean reference signals, traditional reference‐based evaluation metrics, such as SI‐SDR and PESQ, cannot be directly applied in this study. To objectively quantify the model's separation performance, this study adopts Deep Noise Suppression Mean Opinion Score (DNSMOS) (Reddy et al. 2022) as the primary evaluation metric, following related studies (Liao et al. 2025; Tzinis, Adi, et al. 2022). DNSMOS is a non‐intrusive perceptual quality assessment method that does not require clean reference signals and can directly evaluate separated audio. It provides objective estimates of signal quality and residual noise suppression, making it suitable for assessing separated Hainan gibbon calls under real‐world acoustic conditions. DNSMOS provides predictions along three dimensions, with scores typically ranging from 1 to 5. Speech Quality (SIG) evaluates the fidelity and distortion of the target signal, with higher scores indicating that the separated calls closely resemble the natural source in structure and clarity. Background Noise Quality (BAK) measures the effectiveness of noise suppression, with higher scores indicating less residual noise or minimal interference with the target signal. Overall Quality (OVRL) evaluates the overall perceptual quality, integrating assessments of both the target signal and residual noise.

Furthermore, a Hainan gibbon call presence detection task was designed as a downstream evaluation to assess the effectiveness of the separated target‐call estimates. In this evaluation, source separation was applied exclusively to the testing subset and not to the training or validation subsets. The detection models were trained only on preprocessed audio segments without any source separation, whereas separated target‐call estimates were used only as test inputs. Specifically, the trained RemixIT and MC‐RemixIT models were applied to the testing subset of the downstream detection dataset to generate estimated target calls, which were converted into log‐Mel spectrograms and used as inputs to the corresponding downstream detection models. The effects of the different separation approaches were assessed by comparing detection performance under different input conditions.

In the downstream detection experiments, three input conditions were compared, including preprocessed audio segments without source separation, target‐call estimates from RemixIT, and target‐call estimates from MC‐RemixIT. Several CNN architectures were evaluated, including ResNet34 and ResNet50 (Koonce 2021), as well as lightweight architectures such as MobileNetV3s (Howard et al. 2019) and MobileNetV4 (Qin et al. 2024). For each architecture, the same training strategy and experimental settings were maintained across different input conditions. The cross‐entropy loss function and AdamW optimizer were employed, with an initial learning rate of 0.001, a batch size of 32, and training for a maximum of 100 epochs. Performance was evaluated using accuracy (*A*), precision (*P*), recall (*R*), and *F*1‐score (*F*1).

The experimental configuration is shown in Table 1.

**TABLE 1:** Experimental configuration.

Configuration	Parameter
CPU	12 vCPU Intel(R) Xeon(R) Platinum 8352V CPU @ 2.10 GHz
GPU	vGPU‐32GB (32 GB)
Operating system	ubuntu22.04
Accelerated environment	CUDA 11.8
Development environment	Python 3.10
Deep Learning Framework	PyTorch

## 3 Results

To systematically evaluate the effectiveness of the MC‐RemixIT in complex acoustic scenarios, experiments were designed to assess both separation performance and downstream task improvement. Separation performance was evaluated through call and background noise separation experiments on the Hainan gibbon monitoring dataset. The evaluation further investigates the performance improvements contributed by the LCE module, the Signal cross‐correlation constraint, and the multi‐constrained strategy. Furthermore, to assess the practical applicability of the proposed approach for ecological monitoring tasks, Hainan gibbon call presence detection models were constructed using the mainstream ResNet and MobileNet architectures. By comparing changes in metrics such as accuracy and recall before and after separation, the study analyzes the performance gains provided by the proposed method as a front‐end preprocessing module for downstream detection tasks.

### 3.1 Visualization Analysis of Call Separation Results

To visually illustrate the separation performance of the proposed approach on Hainan gibbon calls and background noise, three representative and challenging scenarios from field monitoring were selected for spectrogram‐based analysis.

Figure 4 shows representative spectrograms illustrating the source separation results under low‐SNR field conditions. In complex field soundscapes, target calls are often substantially weaker than the surrounding background noise because of long recording distances or elevated environmental noise levels, as illustrated in the original recordings (Figure 4a,d). For the separated calls, both RemixIT and MC‐RemixIT recover the dominant call components from the mixtures. Compared with the RemixIT (Figure 4b), the call estimates produced by MC‐RemixIT (Figure 4e) exhibit clearer harmonic structures, more continuous syllable contours, and less residual background interference. The estimated noise further highlights the difference between the two approaches. As shown in Figure 4c, the noise estimated by RemixIT contains noticeable residual target‐call components. These residual components are substantially reduced in the noise estimated by MC‐RemixIT(Figure 4f). Overall, the visual comparison suggests that MC‐RemixIT achieves clearer separation between target calls and background noise under low‐SNR conditions.

**FIGURE 4:**
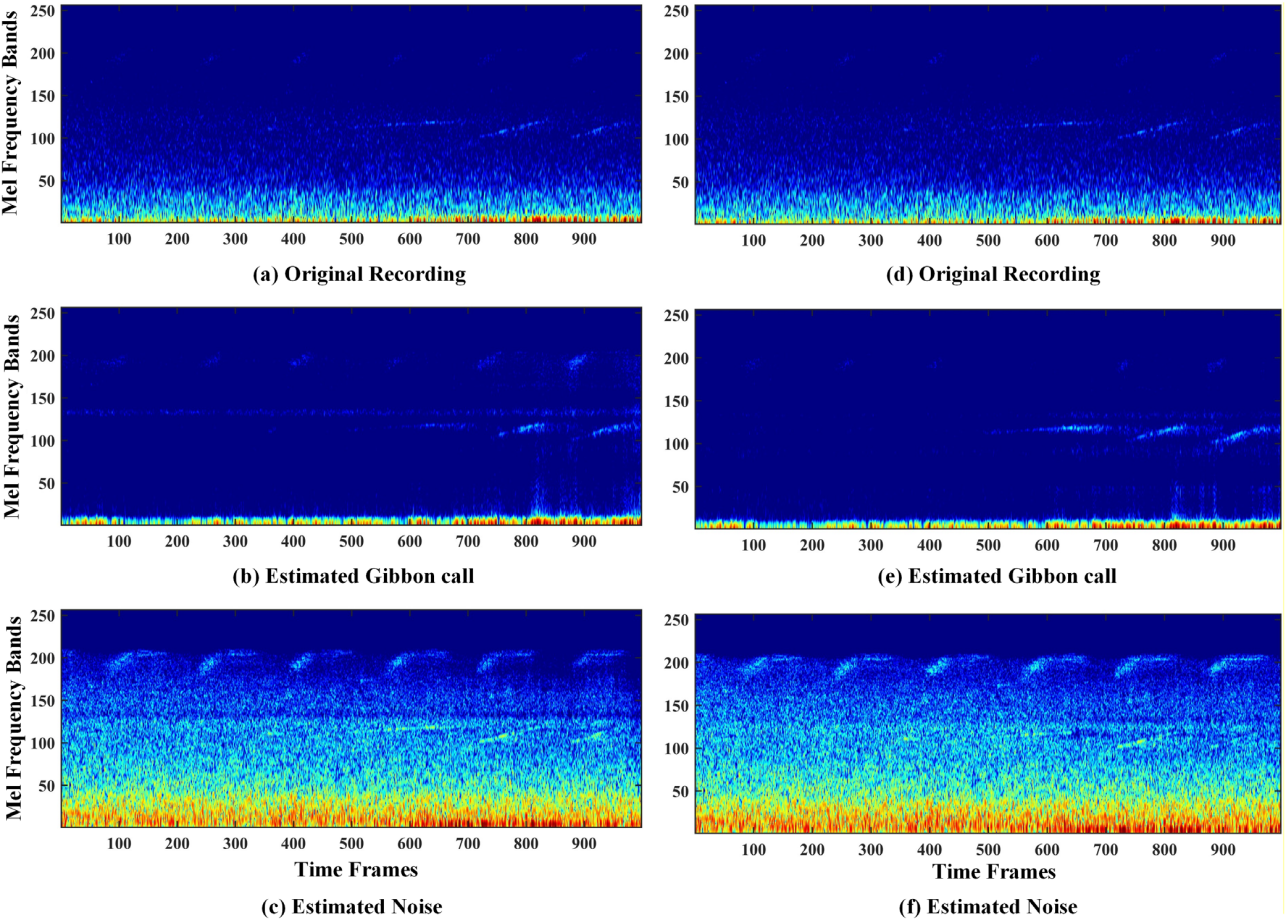
Spectrogram comparison of low‐SNR Hainan gibbon calls recorded in the field. (a, d) Original recordings; (b, c) call and noise estimates from RemixIT applied to (a); (e, f) call and noise estimates from MC‐RemixIT applied to (d).

Figure 5 presents a quantitative evaluation of the call separation results in Figure 4 using DNSMOS metrics. Compared with RemixIT, the proposed MC‐RemixIT consistently achieves performance improvements across all three evaluation dimensions. Specifically, the SIG score increases from 2.82 to 2.93, indicating improved preservation of the target call's structure and clarity. The BAK score for background noise suppression increases from 2.94 to 3.06, suggesting that the proposed model reduces residual environmental noise more effectively. The OVRL increased from 2.57 to 2.63. These results confirm that the quantitative metrics align with the visual observations. MC‐RemixIT simultaneously enhances the perceptual quality of target calls and the effectiveness of noise separation.

**FIGURE 5:**
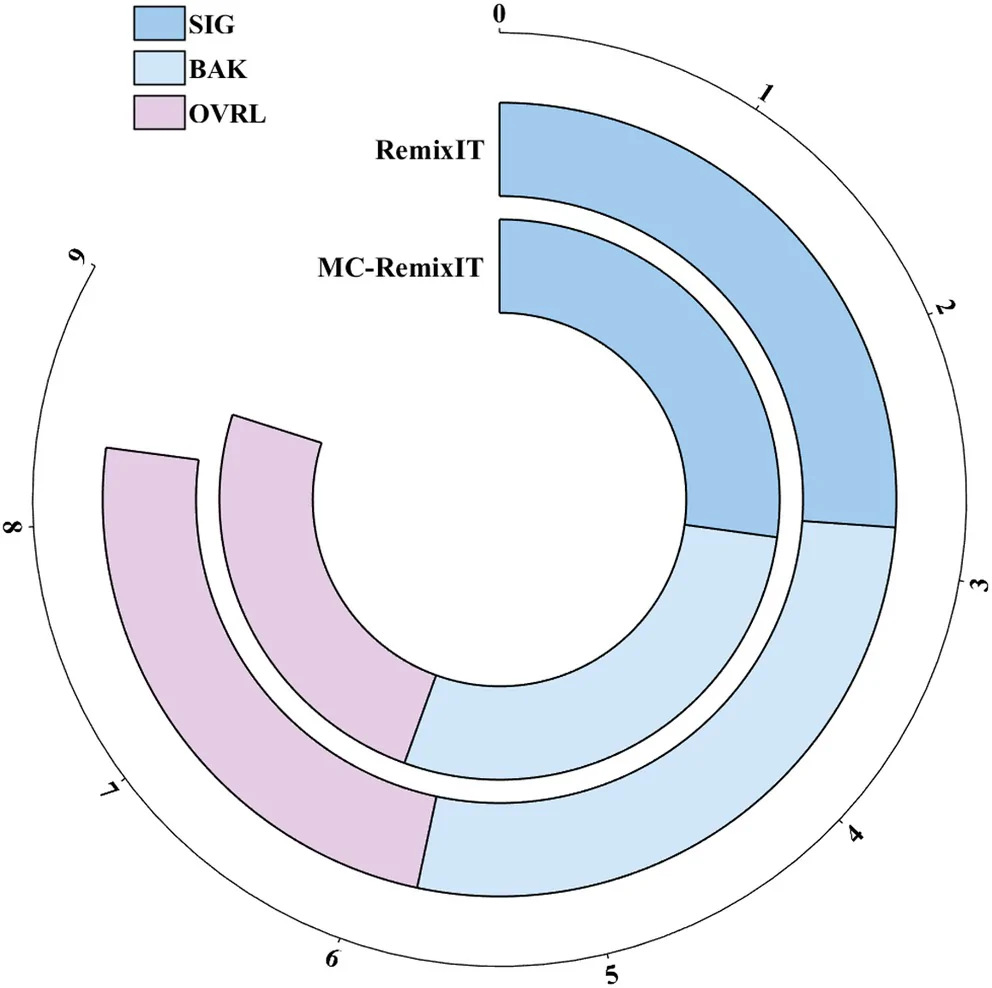
DNSMOS evaluation of call separation using RemixIT and MC‐RemixIT. SIG, BAK, and OVRL denote Speech Quality, Background Noise Quality, and Overall Quality, respectively.

PAM recordings are frequently contaminated by complex background noise, including persistent high‐frequency insect sounds, intermittent calls from other animals, and paroxysmal wind or rain noise. Figure 6 presents the estimated call and noise spectrograms obtained using MC‐RemixIT for three recordings containing different types of background interference. In the original recordings, the time‐frequency structure of Hainan gibbon calls is disrupted by various background sources, as shown in Figure 6a,d,g. The separation results demonstrate that MC‐RemixIT can extract target call features from complex soundscapes, as shown in Figure 6b,e,h. Comparison of the original recordings, estimated calls, and estimated noise shows that the proposed method preserves the dominant calls' structures while effectively separating background noise, demonstrating robust separation performance under complex acoustic conditions.

**FIGURE 6:**
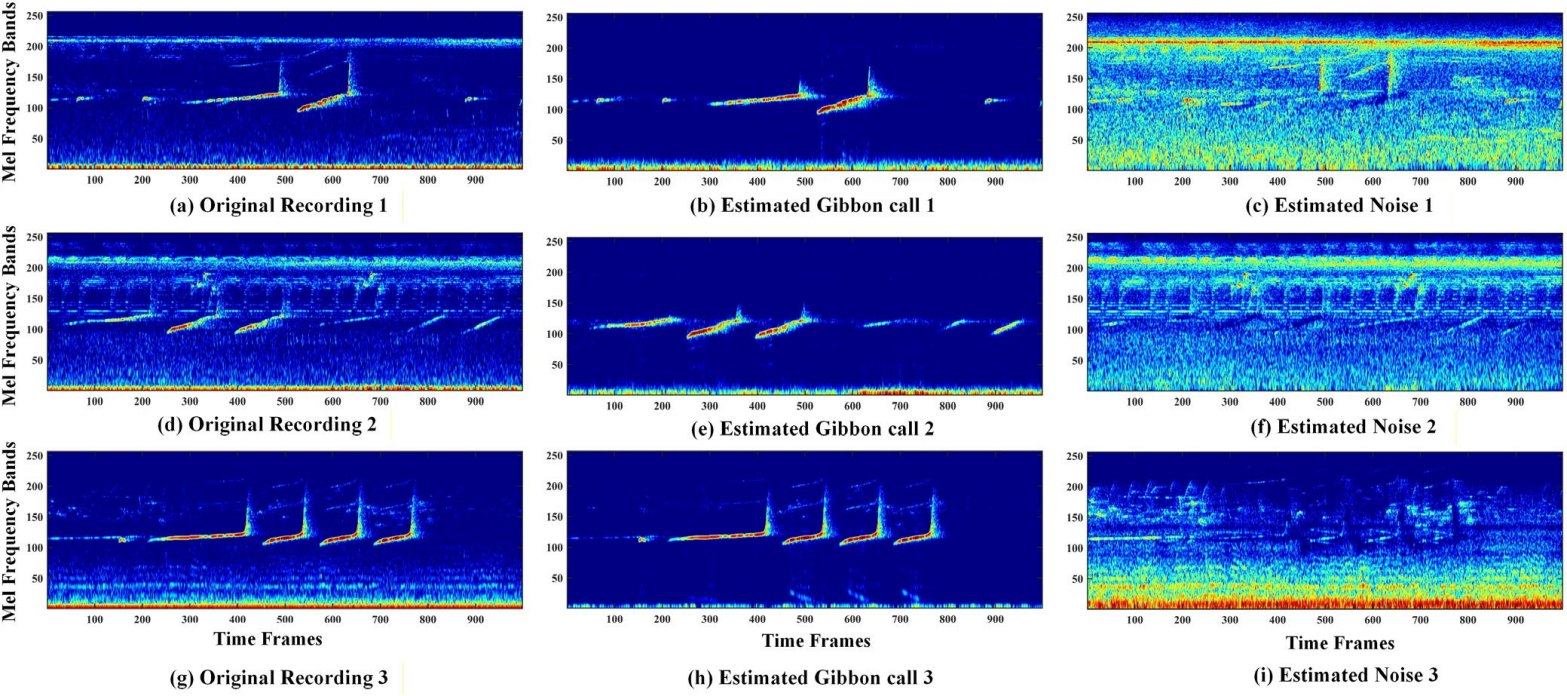
Spectrogram comparison of separation results for Hainan gibbon recordings under various intense noise conditions. (a, d, g) Original recordings, with (d) and (g) containing overlapping calls from two individuals. (b, c), (e, f), and (h, i) show the corresponding estimated call and noise spectrograms for (a), (d), and (g), respectively.

In field monitoring scenarios, recordings often contain simultaneous calls from multiple individuals with significant overlap in both the time and frequency domains. Furthermore, variations in the distance to the recording device and vocalization intensity lead to uneven SNR and energy distributions, with some components exhibiting higher energy while others remain relatively weak, as shown in Figure 6d,g. The separation results obtained by applying the proposed approach to the original recordings in Figure 6d,g are shown in Figure 6e,f,h,i. They demonstrate that, under such complex soundscapes, the method can stably extract target calls while effectively separating noise.

Table 2 summarizes the DNSMOS evaluation results for the three recordings in Figure 6 after separation with RemixIT and MC‐RemixIT. Compared with RemixIT, the proposed approach achieves consistent improvements across all evaluation dimensions. Among all metrics, the most notable improvement is observed in background noise suppression, indicating enhanced reduction of residual environmental noise. The SIG scores of MC‐RemixIT across the three samples show consistent improvement, indicating better preservation of target call structures. The OVRL scores also consistently surpass the baseline, reflecting enhanced overall perceptual quality. The results in Table 2 further validate that MC‐RemixIT maintains a performance advantage under diverse recording conditions, demonstrating its effectiveness in enhancing the perceptual quality of call separation within complex field soundscapes.

**TABLE 2:** DNSMOS results of two recordings after separation using RemixIT and MC‐RemixIT.

Original recording	Evaluation metrics	RemixIT	MC‐RemixIT
Recording 1	SIG	2.64	**2.79**
	BAK	2.79	**3.13**
	OVRL	2.60	**2.64**
Recording 2	SIG	2.75	**2.84**
	BAK	2.89	**3.11**
	OVRL	2.55	**2.63**
Recording 3	SIG	2.80	**2.93**
	BAK	3.00	**3.01**
	OVRL	2.61	**2.76**

*Note:* Bold formatting is used to highlight the best results.

Overall, the proposed method demonstrates effective source separation across a variety of complex soundscapes. It effectively suppresses background interference under challenging acoustic conditions and improves the separation between target calls and noise. In particular, under low‐energy and low‐SNR conditions, the method better preserves the dominant structures of the target calls while reducing residual background interference.

### 3.2 Quantitative Evaluation of Separation Performance

To evaluate the separation performance of MC‐RemixIT, DNSMOS is used to compare it with the baseline RemixIT and the state‐of‐the‐art bioacoustic denoising approach Biodenoising on a Hainan gibbon PAM dataset, as shown in Figure 7. MC‐RemixIT demonstrates consistent advantages across all three perceptual dimensions. Compared with RemixIT, the mean BAK score of MC‐RemixIT increases from 2.03 to 2.32, accompanied by a reduced standard deviation, indicating more stable background noise suppression in complex natural environments. The mean SIG score of MC‐RemixIT is slightly higher than that of RemixIT, indicating improved perceptual quality without noticeable distortion. For OVRL, MC‐RemixIT achieves higher mean and median values with lower variance, further demonstrating its superiority under complex field acoustic conditions.

**FIGURE 7:**
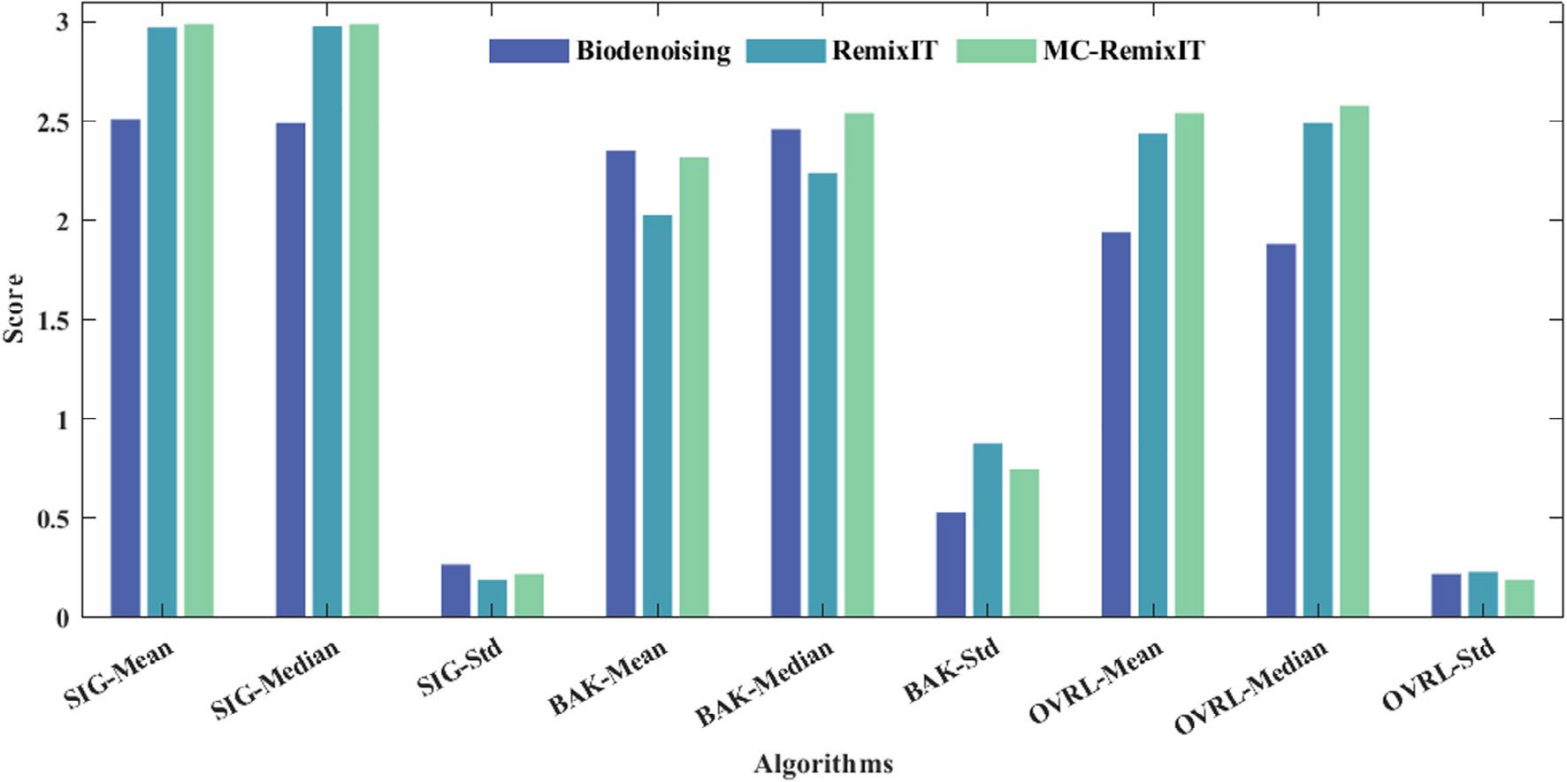
Comparison of separation performance with state‐of‐the‐art models.

Comparison with the Biodenoising further reveals the advantages of the proposed method. Although Biodenoising achieves slightly better background noise suppression, with a mean BAK score of 2.35, it suffers from substantial signal quality degradation, yielding a mean SIG of 2.51 and an OVRL of 1.94, both significantly lower than those of the proposed method. In real‐world ecoacoustic scenarios, MC‐RemixIT achieves a more effective balance between preserving the fidelity of target calls and suppressing background noise. Compared with denoising methods that tend to sacrifice signal characteristics for noise reduction, the proposed model demonstrates a clear advantage in preserving the acoustic features of the target species.

To evaluate the contribution of each module, an ablation study was conducted. The original RemixIT was used as the baseline, and the teacher cross‐correlation strategy (TeaXcorr), multi‐constrained training strategy (MC), student cross‐correlation constraint (StuXcorr), and lightweight call enhancement module (LCE) were progressively incorporated and evaluated using the DNSMOS metric. The results are presented in Table 3.

**TABLE 3:** Ablation experiment.

Model	TeaXcorr	MC	StuXcorr	LCE	OVRL	BAK	SIG
RemixIT	—	—	—	—	2.44	2.03	2.97
+T	**√**	—	—	—	2.49	2.11	2.99
+TM	**√**	**√**	—	—	2.50	2.09	3.01
+TMS	**√**	**√**	**√**	—	2.51	2.17	2.99
MC‐RemixIT	**√**	**√**	**√**	**√**	2.54	2.32	2.99

With the introduction of the TeaXcorr module, all metrics improved, indicating enhanced separation capability and more effective suppression of background noise. Building on this, incorporating the MC increased the SIG score from 2.99 to 3.01, suggesting improved preservation of the structural integrity of the target calls. However, a slight decrease in BAK was observed, indicating a trade‐off between signal fidelity and noise suppression. Adding the StuXcorr increased BAK to 2.17 and improved OVRL to 2.51. Integrating the LCE resulted in both BAK and OVRL reaching their highest values. These results show that the proposed modules, through their combined effects, effectively improve target call separation from background noise while preserving call fidelity as much as possible.

### 3.3 Downstream Detection Task

To further evaluate the effectiveness of the proposed separation framework for downstream detection, a Hainan gibbon call presence detection experiment was conducted. Three input conditions were compared, including preprocessed audio segments without source separation, target‐call estimates generated by RemixIT, and target‐call estimates generated by MC‐RemixIT. The influence of different separation methods on downstream call detection performance was then evaluated.

Figure 8 illustrates the performance of four mainstream deep learning models on Hainan gibbon call presence detection under different input conditions. On the overall test set, the models generally achieved improved accuracy, precision, recall, and *F*1‐score when using target‐call estimates generated by RemixIT or MC‐RemixIT compared with preprocessed audio segments without source separation. These results suggest that the separated target‐call representations provide more effective inputs for downstream Hainan gibbon call detection. Among the three conditions, MC‐RemixIT achieved better overall detection performance across all evaluated models. For the ResNet series, MC‐RemixIT increases the accuracy of ResNet50 and ResNet34 from 80.60% and 81.84% to 90.80% and 88.31%, respectively, with *F*1‐scores reaching 91.31% and 88.84%. A larger relative improvement was observed for the lightweight networks. For example, the accuracy of MobileNetV3s increased from 76.62% without source separation to 88.56% with MC‐RemixIT‐generated target‐call estimates, whereas RemixIT yielded an accuracy of 80.85% for the same architecture.

**FIGURE 8:**
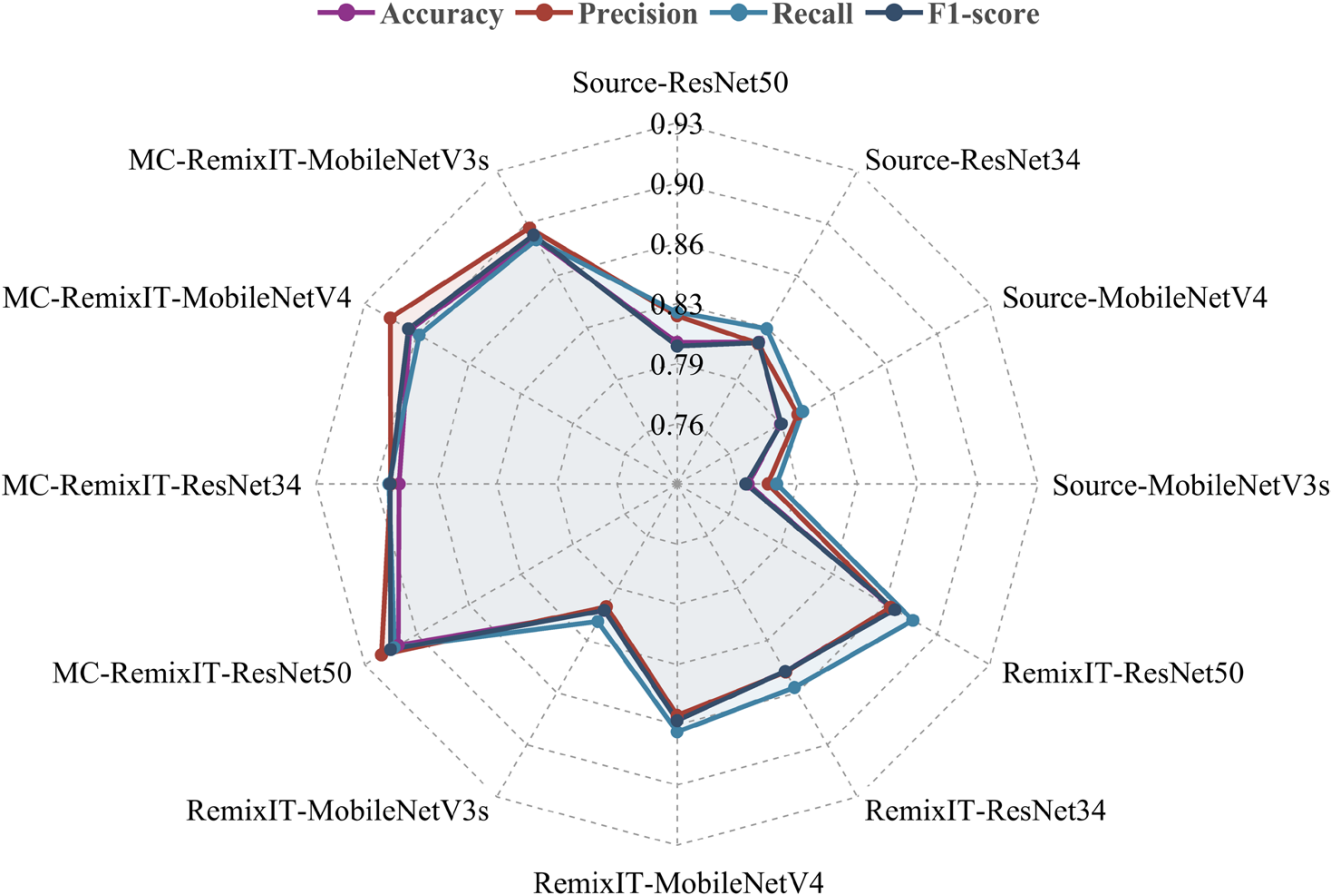
Performance of four deep learning models on Hainan gibbon call presence detection.

To further assess whether the observed performance gains were consistent across different recording sources, the test set of the downstream detection dataset was separated into Dufourq and Wang subsets. Downstream detection performance was then independently evaluated under the three input conditions of Source, RemixIT, and MC‐RemixIT, using the same downstream classification models and evaluation protocol. Precision, recall, and *F*1‐score were computed for each class and then macro‐averaged over the classes present in each test subset. The composition of the two test subsets is detailed in Table S1. The Dufourq test subset contains presence, partial presence, and absence classes, whereas the Wang test subset contains only presence and partial presence classes. The corresponding results are reported in Table 4.

**TABLE 4:** Source‐specific downstream detection performance on the Dufourq and Wang test subsets under three input conditions.

Model	Input	Dufourq			Wang		
		*P* (%)	*R* (%)	*F*1 (%)	*P* (%)	*R* (%)	*F*1 (%)
ResNet50	Source	85.53	83.97	83.36	73.80	60.50	64.70
	RemixIT	86.63	86.83	86.13	86.75	84.65	85.62
	MC‐RemixIT	**91.93**	**91.63**	**91.60**	84.60	80.50	82.21
ResNet34	Source	81.83	81.73	81.26	80.80	79.05	79.85
	RemixIT	85.57	84.97	84.54	83.20	83.20	83.20
	MC‐RemixIT	**90.13**	**90.13**	**90.13**	73.15	65.15	68.08
MobileNetV4	Source	81.43	80.37	79.71	78.50	73.35	75.77
	RemixIT	85.07	85.53	85.11	88.80	88.80	88.80
	MC‐RemixIT	**91.73**	**90.30**	**90.71**	84.45	79.05	81.44
MobileNetV3s	Source	78.27	76.83	75.83	81.65	74.80	78.08
	RemixIT	80.93	80.83	80.49	79.80	72.15	74.59
	MC‐RemixIT	**89.90**	**89.17**	**89.42**	80.65	77.65	79.09

*Note:* Bold formatting is used to highlight the best results.

On the Dufourq test subset, MC‐RemixIT achieved the highest precision, recall, and *F*1‐score across all four downstream classification models, indicating a consistent performance improvement. For ResNet50, MC‐RemixIT achieved precision, recall, and *F*1‐score values of 91.93%, 91.63%, and 91.60%, respectively. Compared with the Source baseline, these values increased by 6.40, 7.66, and 8.24 percentage points. Relative to RemixIT, the corresponding increases were 5.30, 4.80, and 5.47 percentage points. Similar improvements were observed for ResNet34, MobileNetV4, and MobileNetV3s, with MC‐RemixIT achieving *F*1‐scores of 90.13%, 90.71%, and 89.42%, respectively.

On the Wang test subset (*n* = 47), the relative performance of the three input conditions varied across the downstream models. For ResNet50 and MobileNetV4, both RemixIT and MC‐RemixIT achieved higher precision, recall, and *F*1‐score than the Source baseline. For ResNet34, RemixIT outperformed Source, whereas MC‐RemixIT yielded lower values for all three metrics than Source. For MobileNetV3s, RemixIT underperformed Source, whereas MC‐RemixIT achieved higher recall and *F*1‐score than Source.

In summary, MC‐RemixIT consistently improved downstream detection performance across the four evaluated architectures on the overall test set, with a consistent performance advantage on the Dufourq test subset. These results support the effectiveness of MC‐RemixIT as a front‐end separation module for downstream Hainan gibbon call detection. The source‐specific analysis further showed some variation in the relative performance of the three input conditions across recording sources, while MC‐RemixIT maintained a consistent performance advantage on the Dufourq subset. Collectively, these results demonstrate the effectiveness of the proposed separation framework for improving downstream call detection across different detection architectures.

## 4 Discussion

In PAM, Hainan gibbon recordings are frequently contaminated by multi‐source and non‐stationary background noise, while fully clean reference calls required for supervised source separation are rarely available under field conditions. This limitation restricts the application of supervised separation methods for ecological acoustic monitoring. To address this challenge, this study proposed MC‐RemixIT, a self‐supervised source separation framework designed for Hainan gibbon calls. Due to the absence of paired clean references, separation performance was evaluated using DNSMOS‐based perceptual quality assessment. DNSMOS provides a reference‐free evaluation of separated signals by quantifying perceptual signal quality and residual noise suppression, making it suitable for assessing source separation performance under realistic PAM conditions. However, DNSMOS was originally developed for human speech enhancement and may not fully capture species‐specific acoustic characteristics of animal vocalizations. Therefore, DNSMOS alone cannot fully reflect whether separated signals retain task‐relevant acoustic information for Hainan gibbon call recognition. A downstream Hainan gibbon call presence detection task was conducted as a complementary task‐oriented evaluation. The improved detection performance obtained with MC‐RemixIT‐generated target‐call estimates supports the utility of the separated signals for downstream call detection. DNSMOS and downstream detection thus provide complementary perspectives, assessing perceptual separation quality and task‐relevant utility, respectively.

Beyond improving source separation performance, MC‐RemixIT provides practical advantages for PAM applications. In long‐term PAM, the quality of recorded calls directly affects the reliability of subsequent automated analyzes, including call detection, behavioral monitoring, and ecological assessment, yet large‐scale monitoring often involves recordings affected by diverse environmental noise sources. By enhancing the separation of target calls from background noise, MC‐RemixIT improves the quality of acoustic inputs available for downstream analysis. The improved performance observed in the call presence detection task further supports the use of source separation as a front‐end preprocessing step in automated ecoacoustic monitoring under complex recording conditions.

The source‐specific evaluation further showed that the performance advantage of MC‐RemixIT was relatively stable and consistent on the Dufourq recordings, whereas the relative performance of the different separation methods varied to some extent across the downstream models on the Wang recordings. This difference may be related to the composition of the Wang test subset. The subset contained a relatively small number of samples and did not include the absence class, making its macro‐averaged metrics potentially more sensitive to the classification outcomes of individual samples. The size of the Wang test subset was also constrained by the recording‐level partitioning adopted to avoid information leakage between subsets. Beyond sample size and class composition, differences in recording conditions between the two recording sources may also affect the effectiveness of the separated signals in downstream classification. Further evaluation using larger and more class‐balanced datasets covering a wider range of recording conditions would help to more fully assess the applicability of MC‐RemixIT across diverse recording sources.

Species‐specific deep learning models have become increasingly common in wildlife monitoring and conservation. Representative applications include brown bear individual identification (Clapham et al. 2020), giant panda age estimation (Qi et al. 2022), white‐headed langur call detection (Xie et al. 2026), and red‐billed gull automatic identification (Ren et al. 2026). Because monitoring targets, data characteristics, and monitoring tasks vary considerably across species, species‐specific optimization often provides more reliable performance than universal solutions. Accordingly, MC‐RemixIT was specifically designed to separate Hainan gibbon calls in the absence of fully clean reference recordings. Although the current implementation is tailored to Hainan gibbons, the proposed optimization strategy may also provide a useful reference for other species facing similar data constraints. It also provides a practical direction for extending self‐supervised source separation to other PAM applications.

Despite the encouraging performance of MC‐RemixIT, several limitations remain. First, separation performance may decline under extremely low‐SNR conditions, where Hainan gibbon calls are heavily masked by background noise. This limitation reflects the inherent difficulty of distinguishing weak target calls from dominant environmental noise when only limited acoustic information is available. Such conditions are common in long‐term PAM and remain a major challenge for source separation in ecological recordings. Second, the present study focuses on separating target calls from background noise rather than overlapping calls from multiple individuals of the same species. Individual‐level call overlap is frequently encountered in field recordings and remains an important challenge for PAM. Addressing this issue would require methods capable of separating overlapping calls from multiple individuals, which could further expand the utility of the framework for individual‐level and behavioral analyzes.

Overall, MC‐RemixIT provides an effective solution for separating Hainan gibbon calls from complex background noise under realistic PAM conditions, while reducing reliance on fully clean reference recordings that are difficult to obtain in the field. The improvement in separation quality was associated with better downstream call presence detection, indicating that self‐supervised source separation can serve as an effective front‐end preprocessing step for automated ecoacoustic analysis. These findings highlight the value of MC‐RemixIT for long‐term PAM of Hainan gibbons and, more broadly, its potential to improve the quality of acoustic data available for ecological analyzes and conservation research.

## Author Contributions

**Jiali Zi:** formal analysis (equal), methodology (equal), writing – original draft (lead). **Nuo Xu:** data curation (equal), writing – review and editing (equal). **Yanqi Dong:** conceptualization (equal), validation (equal). **Yi Xia:** validation (equal), writing – review and editing (equal). **Weilin Wang:** data curation (equal), formal analysis (equal). **Feixiang Chen:** conceptualization (equal), funding acquisition (lead), methodology (equal), writing – original draft (supporting), writing – review and editing (equal).

## Funding

This work was supported by the National Key R&D Program of China (2022YFF1302700), Outstanding Youth Team Project of Central Universities (QNTD202504).

## Conflicts of Interest

The authors declare no conflicts of interest.

## Supporting information


**Table S1:** Composition of the downstream detection dataset by source, subset, and class.

## Data Availability

Hainan gibbon calls are available from the https://doi.org/10.6084/m9.figshare.24296944 and https://doi.org/10.5281/zenodo.3991714. The code created in the research is available at https://github.com/Aurora‐nl/MC‐RemixIT.git.
