# Supplementary material for: A Self‐Supervised Approach for Hainan Gibbon Call Separation in Passive Acoustic Monitoring
Source: Ecol Evol. 2026 Oct 22;16(10):e74542. doi: 10.1002/ece3.74542 (PMC13688718; doi:10.1002/ece3.74542)
Supplement: Supplementary file 1 [file ECE3-16-e74542-s001.docx]

Table S1. Composition of the downstream detection dataset by source, subset, and class.

| Dataset | Subset | presence | partial presence | absence |
| --- | --- | --- | --- | --- |
| Wang | Train | 679 | 748 | 566 |
|  | Val | 8 | 4 | 0 |
|  | Test | 35 | 12 | 0 |
| Dufourq | Train | 123 | 60 | 237 |
|  | Val | 160 | 144 | 101 |
|  | Test | 122 | 128 | 105 |
